# The effect of different orthodontic mini-implant brands and geometry on primary stability (an in vitro study)

**DOI:** 10.1016/j.heliyon.2023.e19858

**Published:** 2023-09-06

**Authors:** Khanda Latif Abdalla, Trefa Mohammed Ali Mahmood

**Affiliations:** Department of Pedodontics, Orthodontics and Preventive Dentistry, College of Dentistry, University of Sulaimani, Sulaimanyah, Iraq

**Keywords:** Orthodontic mini-implant, Primary stability, Geometry

## Abstract

**Background:**

In orthodontic procedures, mini-implants are routinely used as temporary anchorage devices. Early failure is primarily attributed to a variety of issues, which are mostly connected to the quality and geometry of the screw that lead to insufficient primary stability.

**Objectives:**

To evaluate the primary stability of different sizes and brands of orthodontic mini-implants by optimizing the insertion torque value (ITV) and to clear out which one has the greatest primary stability among the most widely used mini-implants by orthodontists.

**Methods:**

Eighty-two self-drilling mini-implants from three different brands with different sizes were used (Optimus Ortho System (Osteonic made in Korea), Smart anchor (GNI made in Korea) (1.4 × 6, 1.6 × 8 and 1.8 × 10mm) and Morelli (made in Brazil) (1.5 × 6, 1.5 × 8 and 1.5 × 10mm), made from (Ti 6Al 4V). All were drilled at a 60° angle on Sixty artificial bone blocks made from polyurethane foam with a digital torque meter device (Orthonia, Jeil made in Korea), pullout strength (tensile force) was measured with a universal testing machine to find out the best brand and size in the mean of primary stability. Data were analyzed using SPSS Version 25 and JMP Pro Version 16 software using the One-way ANOVA test, the Post hoc and Tukey HCD tests.

**Results:**

There were significant differences between the pullout strength of different sizes for the GNI and OSTEONIC brands, while for the MORELLI brand there were no significant differences between the three different sizes considering ITV (10Ncm) whereas for ITV (20Ncm) there was a significant difference between the different sizes for the pullout of all three brands. GNI was the best brand for all the selected sizes with ITV (10Ncm) and size 1.4 × 6 for ITV (20Ncm), whereas OSTEONIC sizes 1.6 × 8 and 1.8 × 10 were the best for ITV (20Ncm) in term of primary stability.

**Conclusion:**

GNI screws were demonstrated higher primary among the three widely used brands followed by OSTEONIC for size 1.6 × 8 and 1.8 × 10 while MORELLI was the least resistant to dislodgement for the two torque insertion values 10 N/cm and 20 N/cm.

## 1 Introduction

Mini-implants have become one of the most common bone anchorage devices because of their ease of use, patient comfort, and immediate loading capabilities, on the other hand, they have the potential to be loose due to the immediate loading on them [1]. Because of the immediate loading on the mini-implants, primary stability is required, which varies depending on the patient's bone, the design of the mini-implants, and clinical technical factors. It is also referred to as the clinical condition of mini-implant immobility and the ability to resist loads in various directions. To mimic the human cortical and cancellous bone with adequate thickness and mechanical qualities matching to the specific locations of the jaw's in vitro studies, double-layer artificial bone should be used. Mechanical retention between the bone and the mini-implant provides primary stability. Osseo-integration, which occurs as a result of continuous bone remodeling, provides secondary stability for orthodontic mini-implants [2].

For clinicians, selecting the most appropriate geometric screw design is just one of several aspects influencing the stability of mini-implants [3]. The quality (ratio of compact to trabecular bone) and quantity (mineral density) of bone affect the stability of orthodontic mini-implants as well as the mini-implant design parameters. The latter includes variables such as length, diameter, thread depth, width, helix angle and pitch (axial distance between threads), thread depth-to-outer diameter ratio, flutes (recessed areas in the cross-sectional area of the mini-implant that carry bone chips away from the cutting edge as the screw rotates), and body shape (conical, cylindrical) [4]. The length and diameter of the mini-implant are the most critical factors of primary stability [4]. Biological factors related to the quality of bone, oral hygiene, and the forces applied to the mini-implants may affect the stability of orthodontic mini-implants. When other problems that could compromise primary stability are identified, like: low bone density, thin cortical bone, or narrow inter-radicular spaces necessitate the use of small-diameter mini-implants, the optimal geometric design should be chosen. A study done by Walter et al., 2013, confirmed that the outer and inner diameters are the most important factors for primary stability, other design characteristics (cylindrical vs. conical, thread design) may significantly affect primary stability and torsional fracture [5].

The aim of this study was find out the best mini-implant among three widely used brands by orthodontics in term of primary stability which is the most important feature for mini-implants since they are used temporarily for enhancing the anchorage, by evaluating the effects of size and geometry of orthodontic mini-implant on primary stability through optimizing the insertion torque value.

## 2 Material and method

**Fig. 1:**
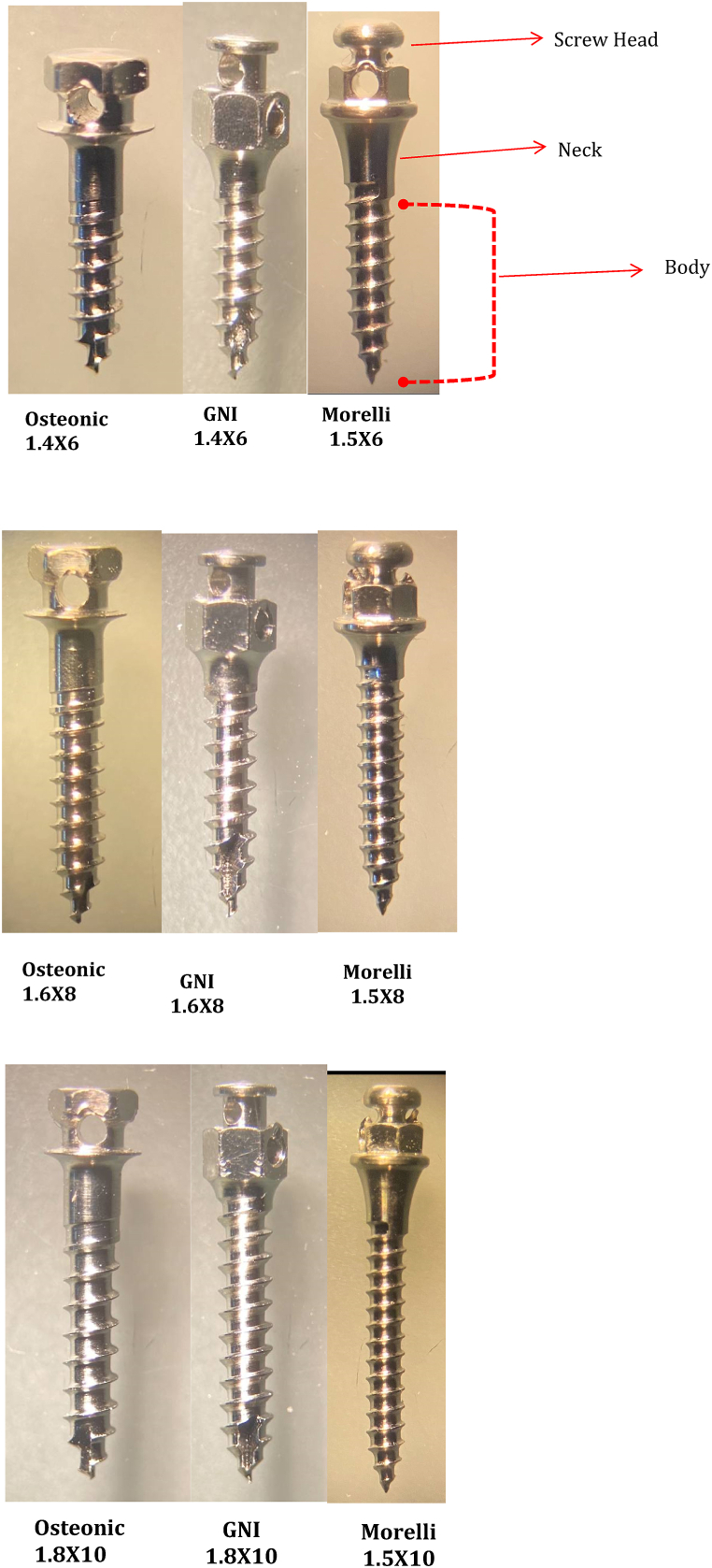
Different brands of screws of the approximately the same size.

Eighty-two self-drilling mini-implants from three different brands with different sizes and designs were used as follows: Optimus Ortho System (commercial name is (Osteonic) made in Guro-gu, Seoul, Korea) (1.4 × 6, 1.6 × 8 and 1.8 × 10 mm) five from each size, Smart anchor (commercial name is (GNI) made in Gyeonggi-do, Korea) (1.4 × 6, 1.6 × 8 and 1.8 × 10mm) five from each size and MORELLI (Morelli is also the commercial name made in Sorocaba state of Sao Paulo, Brazil) (1.5 × 6, 1.5 × 6 and 1.5 × 10mm) four from each size as shown in Fig. 1, pictured with dissecting (stereo) microscope (magnification ratio1×) and they were made from (Ti 6Al 4V).

The sample (Mini-implants).

## 3 Artificial bone blocks

**Fig. 2:**
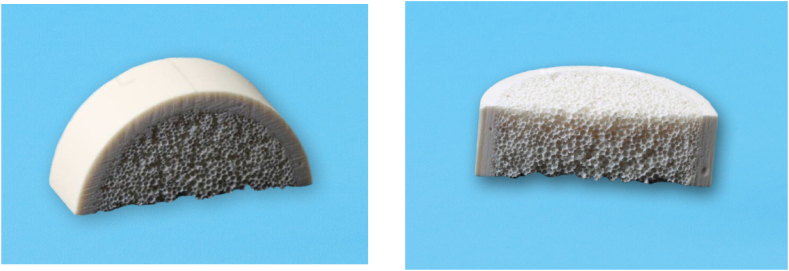
Bone blocks.

Sixty artificial bone blocks (slices) were used; they were constructed from polyurethane foam resembling human bone (Fig. 2). Composed of (cortex and medullary bone), Polyurethane foam is recommended by the American Society of Testing Materials (ASTM) for use in testing mini-implants and other medical devices, having homogenous density and thickness, the use of artificial bones is a substitute for fresh animal bones or human cadaver bones, with a diameter 40 mm, thickness13mm and cortical wall 3 mm, according to Miyamoto et al. the mean cortical thickness of the mandible was 2.22 mm and the mean cortical thickness of the maxilla was 1.49 6 0.34 mm [6,7] with Density 50PCF/0.08 g/cm35.

## 4 Insertion torque measurement

A universal testing machine, CUSSONS TECHNOLOGY LTD (Model Cussons M500-25AT, Manufacture: Great Clowes Street, Manchester, England) with a 100 KN load cell, and a 50 mm gauge length Testometric extensometer (Model Cussons M500-25AT, Fig. 4). was used for testing the pullout strength (tensile force). The machine has 0.001 N accuracy for the force and 0.001 mm accuracy for deformation. The test speed was set at 1 mm/min6, at room temperature (25° Celsius) using Win-Test Analysis software [8] (a universal testing software is a fully-integrated and customizable package that supports all industry standards including ISO, ASTM, and BS EN specifications. Test types supported include tensile, compression, flexure, peel, tear, burst, adhesion, shear, spring, cyclic, friction, and Brinell hardness. It includes a wide range of industry standard test methods and facility to create and store an unlimited number of further test methods. There is automated storage of all test data and ease of export to other software packages such as word, excel, access and SPC systems, made in Germany) where the data is recorded via PC connected to the tester, a fully digital testing system with high precision control and accuracy, includes automated computer control of test methods, Automatic recognition and calibration of load cells and extensometers, with instant calibration check facility and 800% overload capability of load cells without damage (Fig. 4: Universal testing machine).

**Fig. 3:**
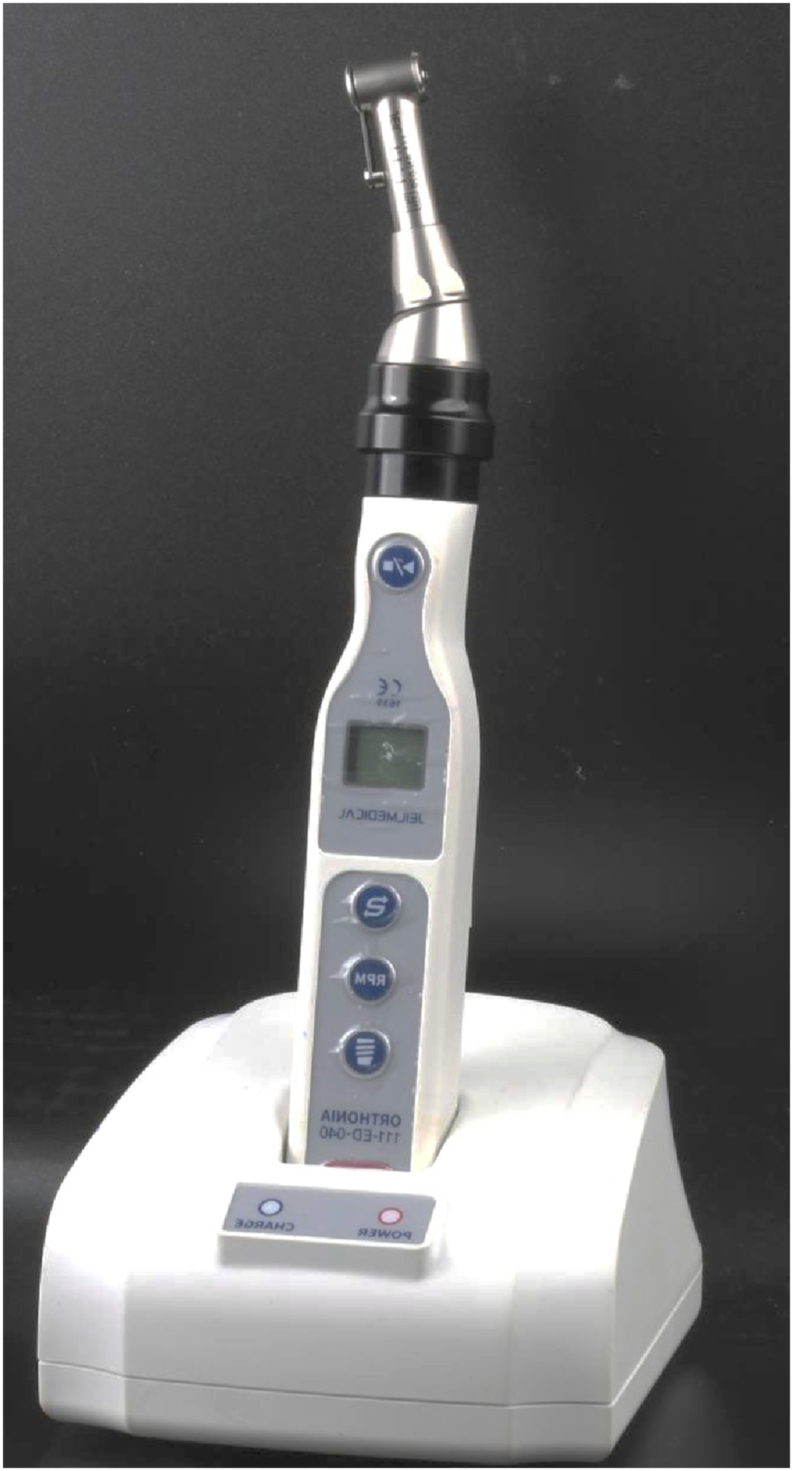
DTM (Orthonia., Jeil) made in Korea.

A Digital torque meter device (DTM from Orthonia, Jeil made in Guro-gu, Seoul, Korea, Fig. 3) was used for drilling, which is a special device either for installing or withdrawing orthodontic mini-screws. The features of having an LCD panel for easier operation, five torque levels every 5 cm from (10 Ncm to 30 Ncm) each with low speed (30 rotation per minute (RPM)), medium (40RPM), and high (50RPM) and having two hand pieces (straight and angle), once the insertion torque installed on the 10Ncm or 20 Ncm the mini-implant will find it's way through the bone block with no pressure, thus these torque values considered as the mean torque value.

**Fig. 4:**
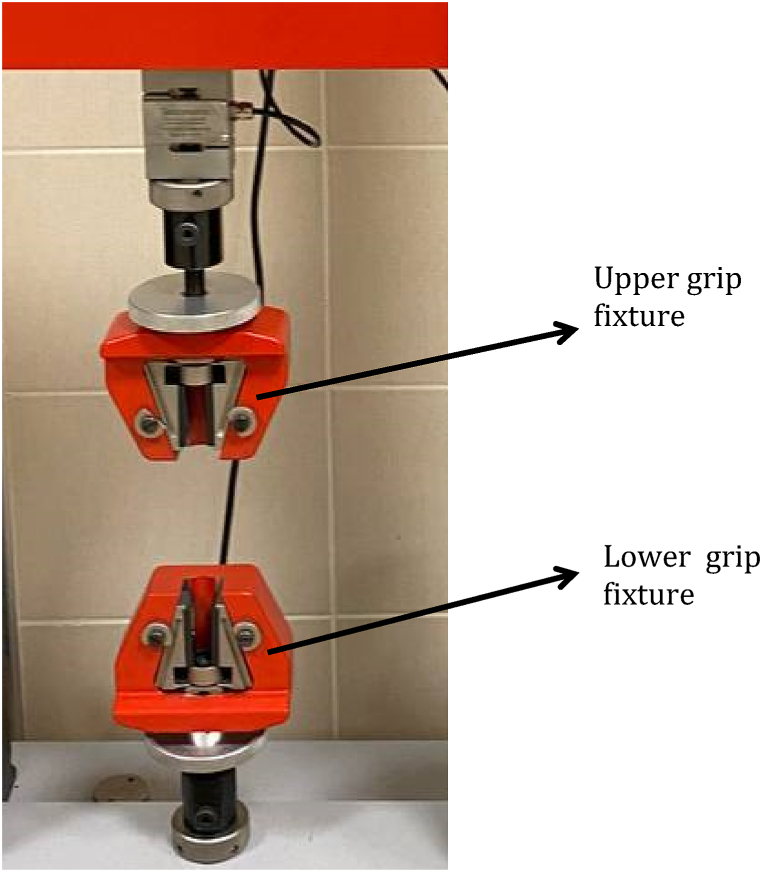
Universal testing machine.

### 4.1 Study protocol

**Fig. 5:**
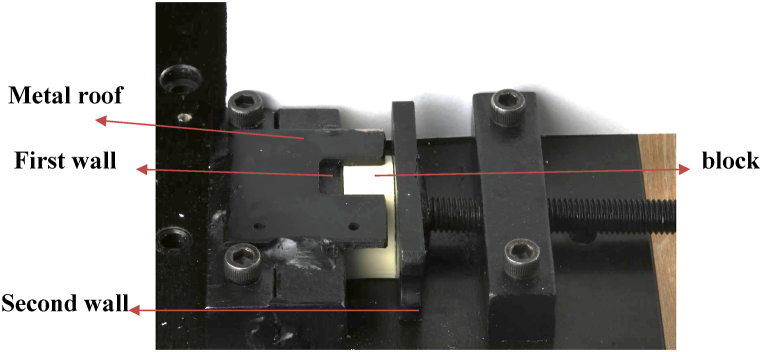
Bone block secure device.

A special metal plate was made with two small walls, one was fixed to the plate which had a roof to secure the bone blocks to prevent its movement in an upward direction and the second wall was attached to a large screw that can be opened to a certain limit to allow for installing the bone-blocks in between the two metal walls (Fig. 5: Bone block secure device).

Each mini-implant was drilled at 60° angle by the same investigator at the same time. Bone blocks (slices) were stabilized with a special custom-made metal secure to stabilize the bone blocks during the insertion of mini-implants by the digital torque meter (DTM).

The insertion torque of the mini-implants was performed by two separate groups; the first group was set at 10 Ncm in medium rotation for every 41 different mini-implants in three brands and three different sizes. The second group was set in 20 Ncm with medium rotation for the other 41 mini-implants with the same brand and sizes as group one.

**Fig. 6:**
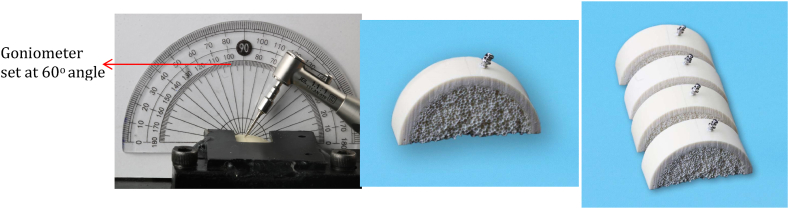
Mini-implant insertion angle using goniometer at 60°.

Each mini-implant was inserted in one bone block, which was marked by a pencil in the middle of its diameter to leave an efficient amount of bone all around the mini-implant. The insertion of the mini-implant into the bone block was performed by the digital torquemeter (Orthonia, Jeil), 2–3 mm away from the marked area, and a goniometer was used to guide the investigator to drill each mini-implant at 60°. The drilling of the mini-implants was continued until all threads were inserted into the bone block without insertion of the neck (collar) part (Fig. 6: Mini-implant insertion angle using goniometer at 60°).

**Fig. 7:**
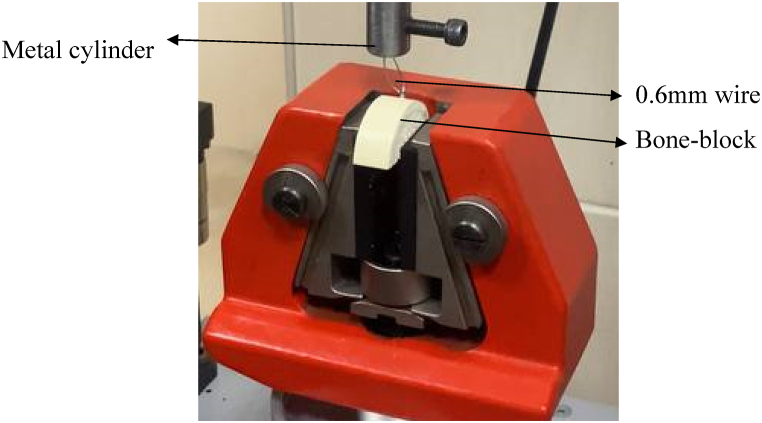
Bone block stabilizer.

After the insertion of each size of mini-implant of all brands, they were ready to measure the pullout force of each by using a Universal Testing Machine (CUSSONS TECHNOLOGY LTD). 0.6 mm round stainless-steel wire (Dentaurum, Ispringen/Germany) was straightened, cut in 10 cm length, and passed through the eyelet of each mini-implant equally (Fig. 7: Bone block stabilizer).

The wire was bent upward all around the eyelet (hole) of the mini-implant and inserted into the hole of a special custom-made metal cylinder that was made for this purpose. A special screw was rotated in a clockwise rotation until the wire was tightened inside the metal cylinder.

The bone blocks were secured in the lower grip fixture of the universal testing machine, while the metal cylinder was secured in the upper grip fixture.

The crosshead speed of the machine was set at 1 mm/min to withdraw the mini-screw from the bone block by applying vertical force parallel to the long axis of the mini-implants [9]. Although this force application in the axial direction is not realistic in clinical use, these tests are commonly used and accepted as a method for comparing mini-implant pullout strengths, which provide valuable information. After the machine and the software were turned on, the lower grip fixture was fixed and the upper grip fixture started to pull the metal cylinder upward automatically at the speed of 1 mm/min, and the stainless-steel wire was pulled upward and the mini-implant was gradually undrilled, the software measured the actual force in which the mini-implants start to deboned from the bone-block that would be the pullout force. This procedure was repeated for each mini-implants in both groups that were 10Ncm and 20Ncm torque groups.

### 4.2 Mini-implants geometry

**Fig. 8:**
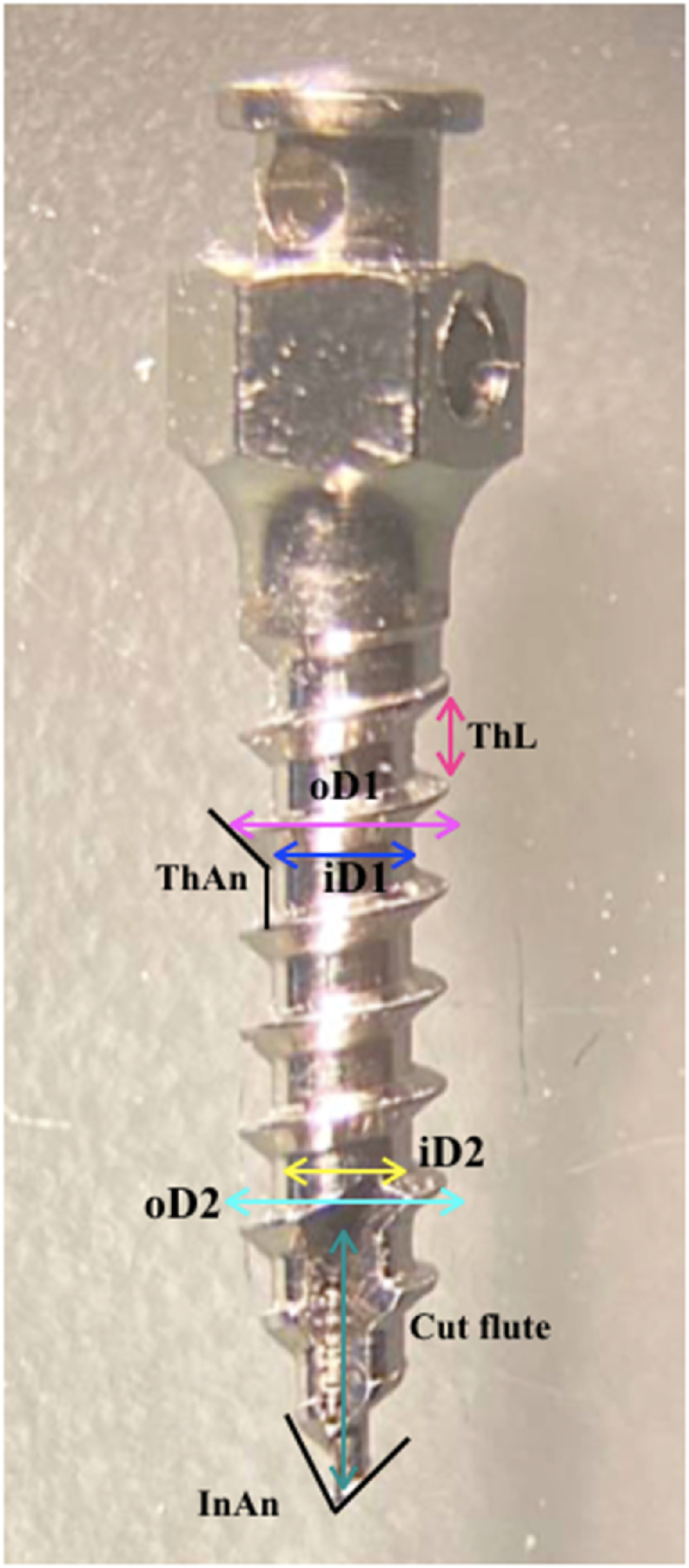
Screw Geometry ThL: Thread length, ThAn: thread angle, iD1: inner thread diameter at the upper limit, iD2: inner thread diameter at the lower limit, oD1: outer thread diameter at the upper limit, oD2: outer thread diameter at the lower limit, Th no: thread numbers, Tht1: Thread thickness at level 1, Tht2: Thread thickness at level 2.

Screws’ geometry was estimated from an image taken by stereo-microscope with a magnification of 1x (dissecting microscope OPTIKA, MS/SFX, Series 20 mm field of view, up to 40x maximum magnification. Highly versatile and appreciated for the 3D viewing. Vertical heads can incline to 45° to give a comfortable posture to the user. Made in Ponteranica (BG)-Italy)as shown in Fig. 8) to find out the following parameters: Thread length (ThL), Thread angle (ThAn), Inner thread diameter at the upper limit (iD1), Inner thread diameter at the lower limit (iD2), outer thread diameter at the upper limit (oD1), Outer thread diameter at the lower limit (oD2), Thread numbers (Th no), Thread thickness at level 1 (Tht1), Thread thickness at level 2 (Tht2) and the images were analyzed via Fiji (ImageJ) software after calibration by setting the scale to uM (mm = 10μM) for standardization.

### 4.3 Statistical analysis

To determine sample size; JMP Software Package was used as shown below.

**Figure:**
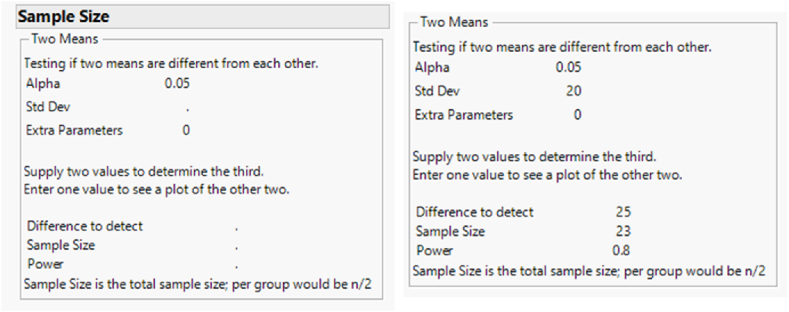

Alpha equal to 0.05 as a significant level, and standard deviation of 20 between the two groups, and a power of 0.80. The total number of samples for both groups is 23, which means that (23/2) which is (11.5) mini-implants is required for each group.

All the data were normally distributed (as shown in Table 1) using Shapiro-Wilk test as the *(Prob* < *W)* , and it is more than α=0.05.

**Table 1:** Shows the goodness-of-fit test (data normality test) for GNI TORQUE 10.

Brand	Shapiro-Wilk W Test			
	TORQUE 10		TORQUE 20	
	W	Prob < W	W	Prob < W
GNI	0.9586138	0.7002	0.9412881	0.4351
MORALLI	0.9725469	0.9356	0.9326432	0.4090
OSTEONIC	0.9503369	0.5298	0.9174705	0.1762

Data were analyzed using SPSS Version 25 and JMP Pro Version 16 software; mean, standard deviation, minimum and maximum values illustrating torque insertion and pullout strength. One-way ANOVA test was used to do a hypothesis test for the different size groups of the same brand in each torque 10 and 20 to determine whether there is a significant difference in the mean scores of their pullout forces and the Post hoc and Tukey HCD tests were then used for multiple comparisons of pullout force between size groups. The correlation coefficient and color map were used to see the relations between the same size groups in pullout force between the different brands of torque (10 and 20). In addition, a one-sample *t*-test was used for the pullout force in different size groups of torque 10 and 20 to determine the best size and brand among them.

## 5 Results

The mean score for the pullout force of the GNI of the size (1.4 × 6), (1.6 × 8), and (1.8 × 10) is 181.3 ± 20.8, 218.2 ± 26.2 and 236.9 ± 26.3 respectively. Their mean scores are far from each other as shown in Table 2. The mean score for the pullout force of OSTEONIC of the sizes (1.4 × 6), (1.6 × 8) & (1.8 × 10) were 128.4 ± 32.3, 197 ± 9.17, and 212 ± 38.7 respectively. And the mean score for the pullout force of the MORELLI of the size (1.5 × 6), (1.5 × 8), and (1.5 × 10) were 145.95 ± 11, 157.7 ± 23.32 and 171.85 ± 19.3 respectively dealing with iTq 10.

**Table 2:**
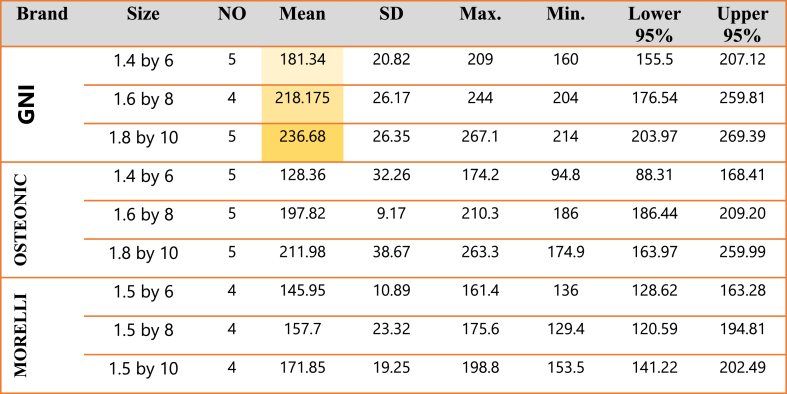
Means and Standard Deviations for the pull-out force of Insertion Torque 10 of the GNI, OSTEONIC & MORELLI.

Descriptive statistics for the pull-out force of iTq 20 of the three brands was shown in Table 3, confirming that GNI has the highest resistance to displacement.

**Table 3:**
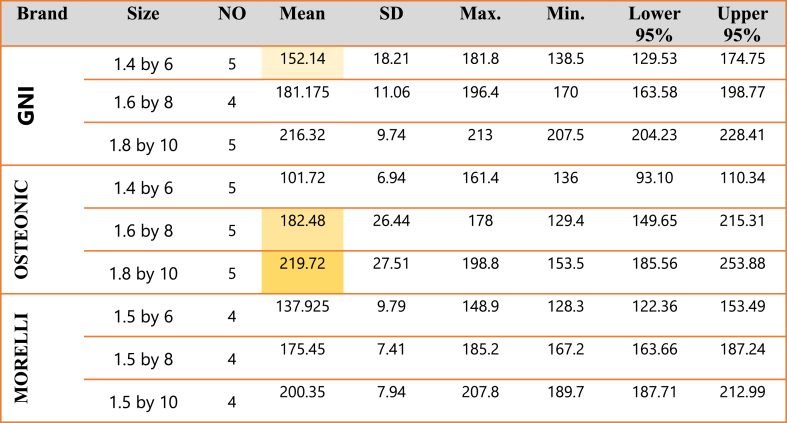
Means and Standard Deviations for the pull-out force of Insertion Torque 20 of the GNI, OSTEONIC & MORELLI.

There were significant differences between the means of the different sizes for the pullout force of the GNI and OSTEONIC brand while for the MORELLI brand there were no significant differences between the three sizes considering torque 10 while for torque 20 there were significant differences between the means of the different sizes for the pullout force of the three brands as in Table 4.

**Table 4:** One-way Analysis of Variance (One-way ANOVA) for pull-out force of the GNI, OSTEONIC & MORELLI with Insertion Torque 10 and 20.

iTq	Brand	Source	Mean Square	p-value
10	GNI	Size	3948.14	0.0130*
		Error	596.73	
		C. Total		
	OSTEONIC	Size	10014.6	0.0016*
		Error	873.3	
		C. Total		
	MORELLI	Size	672.730	0.1974
		Error	344.347	
		C. Total		
20	GNI	Size	5162.17	0.0001*
		Error	188.42	
		C. Total		
	OSTEONIC	Size	18194.2	0.0001*
		Error	501.3	
		C. Total		
	MORELLI	Size	3950.01	0.0001*
		Error	71.24	
		C. Total		

**Fig. 9:**
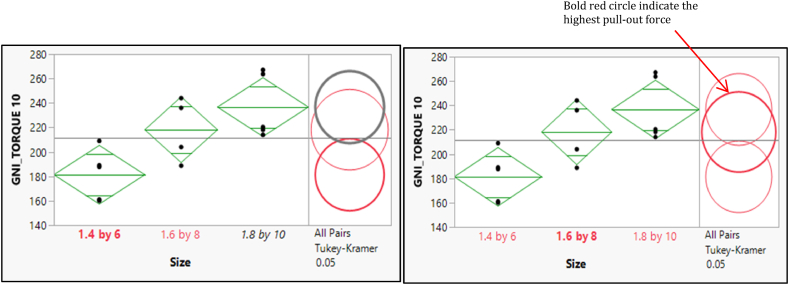
Post hoc of Means Comparisons for the different sizes of the pull-out force of GNI Torque 10.

A significant difference between the two pairs of means was illustrated in Fig. 9. The selected mean had a bold, red circle and a variable label. The mean was significantly different from the selected mean and have a gray circle and gray italicized variable labels. In contrast, the circle's red color and variable labels indicate no difference between them.

**Fig. 10:**
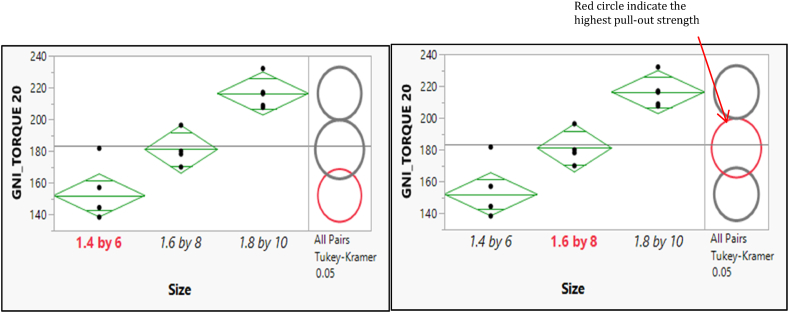
Post hoc of Means Comparisons for the different sizes of the GNI Torque 20.

**Fig. 11:**
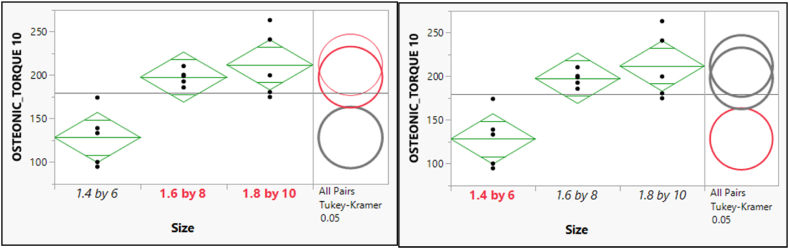
Post hoc of Means Comparisons for the different sizes of the OSTEONIC Torque 10.

**Fig. 12:**
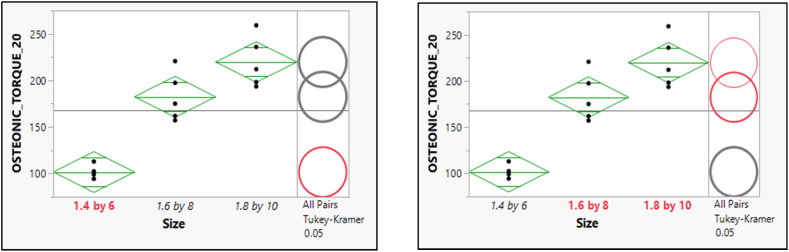
Post hoc of Means Comparisons for the different sizes of the OSTEONIC Torque 20.

The post hoc of means comparisons for different sizes groups which shows a significant difference between the two pairs of means as shown in Fig. 10. As a result, the mean for the GNI Torque 20 with size 1.4 × 6 is statistically significantly different from the mean for both sizes of (1.6 × 8) and (1.8 × 10). Furthermore, the size (1.6 × 8) shows a significant difference from the size (1.8 × 10). Fig. 10 depicts the post hoc of means comparisons to illustrate the significant difference between the two pairs of means. Consequently, the mean for the OSTEONIC Torque 10 with size (1.4 × 6) is statistically significantly different from the mean for both sizes of (1.6 × 8) and (1.8 × 10) and vice versa. In contrast, the size (1.6 × 8) shows an insignificant difference from the size (1.8 × 10). Fig. 11 shows the post hoc analysis of mean comparisons to demonstrate how different the two pairs of means are. As such, the mean score for the OSTEONIC torque 20 with a size of (1.4 × 6) is statistically different from the mean score for (1.6 × 8) and (1.8 × 10), and vice versa. On the other hand, there is no difference between (1.6 × 8) and (1.8 × 10) as in Fig. 12.

**Fig. 13:**
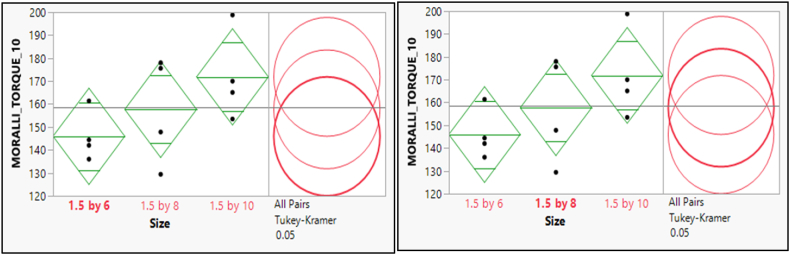
Post hoc of Means Comparisons for the different sizes of the pull-out force of MORELLI Torque 10.

**Fig. 14:**
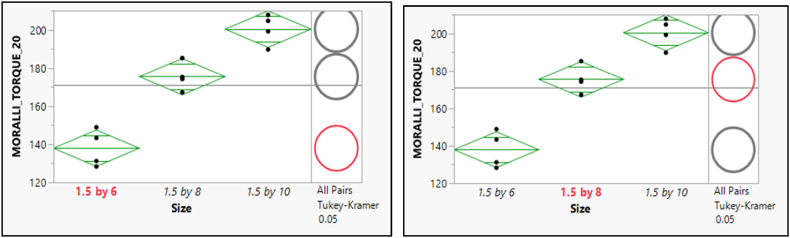
Post hoc of Means Comparisons for the different sizes of the pull-out force of MORELLI Torque 20.

There was a difference between the two pairs of means for the pullout force of MORELLI Torque 10, Furthermore; there is a significant difference between the mean scores of sizes (1.5 × 8) and (1.5 × 10) as in Fig. 13, Fig. 14.

**Fig. 15:**
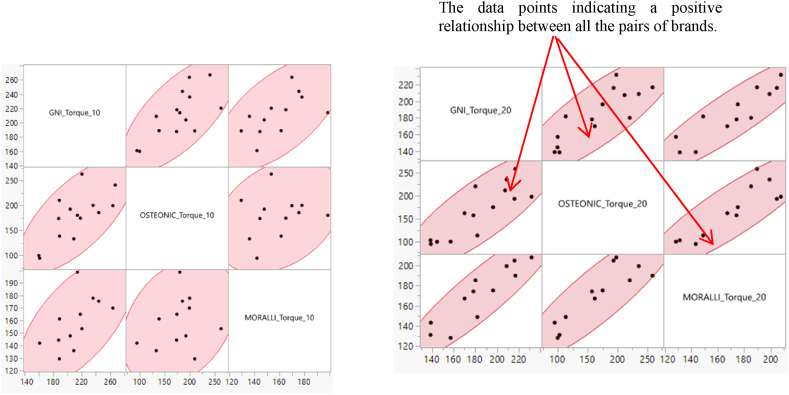
Scatterplot matrix of the correlation between the different brands in torque 10 and 20.

The correlations between GNI, OSTEONIC, and MORELLI brands for pullout force in Torque 10 and 20 were shown in Fig. 15, the data points move toward the origin point, indicating a positive relationship between all the pairs of brands.

The p-values of the pairwise variables (OSTEONIC, GNI) and (MORELLI, GNI) are 0.0052 and 0.046, which are less than 0.5, there is evidence to reject the null hypothesis, and the correlation between these two pairs of brands is statistically significant. In other words, there is a linear correlation between these pairwise brands (Table 5).

**Table 5:** The pairwise correlation of the pull-out force for the different brands in torque 10.

Variable	by Variable	Correlation	Count	Lower 95%	Upper 95%	p-value	−.8 -.6 −.4 -.2 0 .2 .4 .6 .8
OSTEONIC	GNI	0.7017	14	0.2727	0.8980	0.0052*	
MORELLI	GNI	0.5845	12	0.0159	0.8674	0.0460*	
MORELLI	OSTEONIC	0.1797	12	−0.4395	0.6831	0.5763	

In contrast, the lower-level correlation of the pairwise of MORELLI and OSTEONIC is negative (−), and the upper-level correlation is positive (+), zero is a value between these two levels. It indicates that the correlation between these two brands might become zero. That is why the correlation of pullout force between these two brands is not significant as in Fig. 15.

The p-values of the pairwise variables (OSTEONIC, GNI), (MORELLI, GNI), and (MORELLI, OSTEONIC) are 0.0002, 0.0001, and 0.0002, respectively. Since all the p-values are less than α = 0.05, there is evidence to reject H0, and the correlation between these pairs of brands is statistically significant (Table 6).

**Table 6:** The pairwise correlation of the pull-out force for the different brands in torque 10.

Variable	by Variable	Correlation	Count	Lower 95%	Upper 95%	p-value	−.8 -.6 −.4 -.2 0 .2 .4 .6 .8
OSTEONIC	GNI	0.8377	14	0.5528	0.9473	0.0002*	
MORELLI	GNI	0.9027	12	0.6821	0.9727	<.0001*	
MORELLI	OSTEONIC	0.8773	12	0.6109	0.9652	0.0002*	

The geometry of the three brands that were used was described in Table 7, illustrating the shape and size by comparing thread diameter at two different levels, the number, length, thickness, and angle of threads in addition to the cutting angle value since the primary stability is influenced by the shape and geometry.

**Table 7:**
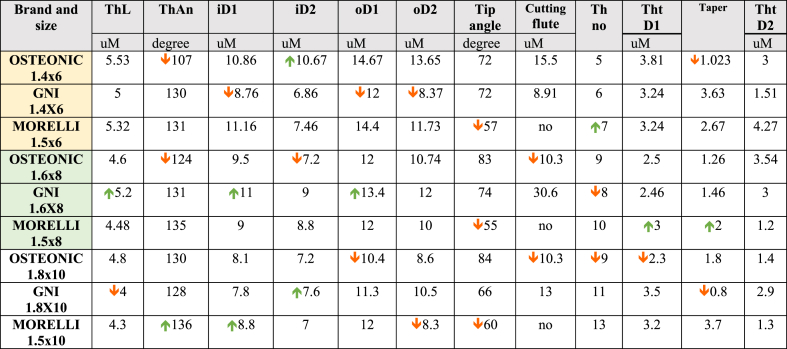
The Geometry of different brands and sizes of the screws. ThL: Thread length, ThAn: thread angle, iD1: inner thread diameter at the upper limit, iD2: inner thread diameter at the lower limit, oD1: outer thread diameter at the upper limit, oD2: outer thread diameter at the lower limit, Th no: thread numbers, Tht1: Thread thickness at level 1, Tht2: Thread thickness at level 2.

## 6 Discussion

Mini-implants have recently become more widely used in orthodontics to prepare orthodontic anchoring in clinical patients. In contrast to studies on prosthodontics implants, investigations on the stability of orthodontic mini-implants are still in their early phases. Design, placement techniques, amount, and quality of the bone, are all factors that affect primary stability [10]. No previous study was done comparing primary stability of different mini-implant brands that were tested on artificial bone block (slice) resembling human bone (cortex and medullary bone) with the use of digital torque meter device. However; studies like Florvaag et al., 2010, Wilmes and Drescher 2011 and Pithon et al., 2012 used different maxillary and mandibular regions of swine, pig bone and Bovine femoral heads respectively, besides in a study done by Florvaag et al., 2010; the drilling was done at different angles 20 and 40° not 60° in which was used in the our study since it was found to be the optimum insertion angle recently; and not including the brands that was addressed in the present study. While; Yashwant et al., 2017, used artificial bone block but they addressed the thread type not the size and the geometry of the mini-implants [[11], [12], [13], [14]]. Using animal bone had the limitation of different bone density and geometry, which may lead to loss of the standardization of bone-screw interface. Using a digital torque meter device to drill in the artificial bone blocks following a standardized protocol is the only way to eliminate the confounding factors related to the drilling criteria that may effect the efficiency of mini-implants in terms of primary stability, since manual drilling or even motor drilling without controlling the TIV and the speed of rotation per minute will dramatically affect the values of pullout strength of the mini-implants, as this study is confirming.

The most common parameter used for quantifying the stability of mini-implants is the pullout strength [15]. That is why this research centered on the size and design of the mini-implant, which is a mechanical behavior that relates to primary stability. In this study eighty-two mini-implants used in two different torque groups (10 and 20Ncm), different sizes and brands each with a different geometrical design were drilled in artificial bone blocks, three brands were selected among the most widely used by orthodontist in Kurdistan region because of their low cost and availability in the market. ASTM recommends polyurethane foam for testing mini-implants and other medical devices. Especially, by controlling density and thickness, the use of artificial bone as a substitute for fresh animal bones or human cadaver bones is a great option. 60° was the drilling angle, according to previous studies assuming the highest primary stability was measured when the insertion angle was between 60° and 70° [16,17]. The primary stability was measured by measuring the pullout force (POF) of each mini-implant while they are withdrawn by Universal Testing Machine device [18,19]. These results show that the primary stability in each brand increases while their sizes are increasing, having the highest (POF) in the largest size (diameter and length), which is (1.8 × 10) for GNI and OSTEONIC, and (1.5 × 10) for MORELLI. Ye et al., 2022 concluded that there was no mini-implant damage or peripheral bone fracture when the ITV varied from 3.28 to 14.65 N cm. thus 10 N cm and 20 N cm were used as ideal torque insertions. The results for 20Ncm insertion torque were the same as torque 10Ncm, in which the (POF) increased by increasing mini-implant size. The results from our study are close to other studies like Wu et al., 2011 that the POF was (109.72, 133.14, 139.68) Ncm for three different brands (Abso Anchors, Bioray, and Lomas) respectively. Another study the result of POF were (195.0,190.7193.9) for three different material and sizes; (1.5 × 10 mm, titanium alloy), (1.5 × 10 mm, stainless steel), and (1.5 × 9 mm, titanium alloy) respectively [20]. Furthermore, the mean score of the POF in the GNI brand size (1.4 × 6) is 181.34, (1.6 × 8) is 218.175, and (1.8 × 10) 236.68 which is the largest mean score compared to other brands with the same sizes with a p-value of 0.0001. So GNI has the highest score of POF among other brands in torque 10 in all sizes. The results are quite different in torque 20Ncm between different brands showing that the POF for the GNI brand in size (1.4 × 6) is 152.14 having the greatest value indicating that GNI brand in torque 20 size group of (1.4 × 6) has the highest primary stability among other brands with the same size groups, but in size (1.6 × 8) and (1.8 × 10) the OSTEONIC brand scores 182.48 and 219.72 respectively, recording the highest score among others. MORELLI brand had the lowest score of POF among all three brands recording the lowest primary stability. As mentioned before, the POF is not the only factor that affects primary stability, factors such as bone thickness and density, the effect of roots, soft tissue, types and amount of orthodontic force, and jaw movement are also related to primary stability of mini-implants in which they were not taken into account in our in vitro study, this is a limitation in our study which can be taken into consideration for future researches. On the other hand, the design, geometry, and size of the mini-implant have a great effect on primary stability, which is a part of our study.

A limitation of the current study was the use of artificial bone blocks which could not allow for investigation of real bone properties at the mini-implant interface [21] and absence of the soft tissue mobility specially at free mucosal area that may reduce the primary stability. However, accurate evaluations to draw a clear conclusion cannot be done with the use of real bone because of the different bone density and geometry providing individual variations. As the current study focused on evaluation of primary stability of different mini-implant brands thus eliminating the confounding factors was an issue to obtain well-controlled experimental conditions. Additionally, future studies should be conducted about other important aspects, such as mini-screw diameter, geometric design and damages to surrounding tissues [[22], [23], [24]].Considering the geometry for GNI and OSTEONIC; the construction of a cutting flute may be responsible for increasing the resistance to dislodgment by increasing the efficiency of drilling through the bone thus reducing the bone fragments and heating, Beside the cutting angle is less acute in the MORELLI screws for all sizes; maybe the construction of MORELLI in this geometry is for increasing the cutting efficiency but unfortunately, this led to reducing the primary stability because of the absence of cutting flute. Besides, within the same size, the number of threads in OSTEONIC is less than that of GNI & MORELLI respectively, however, this did not help increase the primary stability.

The thread length for all of the screws was approximately the same, the upper limit thread diameter in the OSTEONIC screw was more than that of GNI & MORELLI screws, when comparing the upper thread diameter and lower threat diameter between the three brands it was clear that GNI was the least taper one while MORELLI was the most tapered one. So, the Geometry and the design were more favorable in the GNI & OSTEONIC in terms of primary stability compared with MORELLI screws.

The vast majority of studies have demonstrated that primary stability is largely influenced by the surface to which the implant is applied and by the implant-bone interface itself, which is in turn greatly influenced by the implant design. Primary stability is not as strongly correlated with implant length as much as it is correlated to the diameter. The body shape of the implant (cylindrical or tapered), diameters, thread depths, thread diameter, taper angles, and thread pitch are among the properties that affect primary stability. The selection of the mini-implant design and size is essential to establishing adequate primary stability, depending on the site of insertion and local bone quality [17].

Using a digital torque meter device to drill in the artificial bone blocks following a standardized protocol is a way to eliminate the confounding factors related to the drilling criteria that may effect the efficiency of mini-implants in terms of primary stability, since manual drilling or even motor drilling without controlling the TIV and the speed of rotation per minute will dramatically affect the values of pullout strength of the mini-implants creating a bias in the results.

And apart from the fact that mini-implants are widely used in orthodontics, the market explosion with different types, brands, sizes, and geometries often leads to confusion as to which one is the best choice for preventing fracture or dislodgement, which may lead to further exposing the patient to the procedure of drilling, pain, and even the need for the orthodontist to shift to less favorable areas for drilling in terms of patient discomfort (more posterior) and less suitable biomechanics from the point of force application and direction.

## 7 Conclusion

GNI screws were demonstrated higher primary among the three widely used brands in term of primary stability, followed by OSTEONIC for sizes 1.6 × 8 and 1.8 × 10 while MORELLI was the least resistant to dislodgement for the two torque insertion values 10 N/cm and 20 N/cm.

## Funding statement

This work did not receive any specific grant from funding agencies in the public, commercial, or not for profit sectors.

## Ethics approval statement

The ethical committee at the College of Dentistry/University of Sulaimani gave their approval for this study (approval number: 73/21 on 9th Nov.2021).

## Author contribution statement

Khanda Abdalla and Trefa Mahmood: Conceived and designed the experiments; Performed the experiments; Analyzed and interpreted the data; Contributed reagents, materials, analysis data and wrote the paper.

## Data availability statement

Data will be made available on request.

## Declaration of competing interest

The authors declare that they have no known competing financial interests or personal relationships that could have appeared to influence the work reported in this paper.
